# Compressibility and High-Pressure Structure of CaMg_2_Bi_2_ and YbMg_2_Bi_2_

**DOI:** 10.1021/acs.inorgchem.6c02769

**Published:** 2026-09-22

**Authors:** Mario Calderón-Cueva, Allison Pease, Cheng Peng, Wanyue Peng, Megan Rylko, Weiwei Xie, Susannah M. Dorfman, Alexandra Zevalkink

**Affiliations:** † Chemical Engineering and Materials Science Department, 3078 Michigan State University, East Lansing, Michigan 48824, United States; ‡ Department of Earth and Environmental Sciences, Michigan State University, East Lansing, Michigan 48824, United States; § Chemistry Department, Michigan State University, East Lansing, Michigan 48824, United States

## Abstract

Compounds with the formula *AM*_2_ *X*_2_ in the CaAl_2_Si_2_ structure type have garnered increasing interest across various solid-state research domains, such as quantum topological and thermoelectric materials. Prior studies have identified high-pressure phase transitions in several compounds, including Mg_3_Sb_2_, Mg_3_Bi_2_, CaMn_2_Bi_2_, and SrAl_2_Si_2_. In this study, we investigate the structural behavior of CaMg_2_Bi_2_ and YbMg_2_Bi_2_ under varying pressure conditions. We synthesized crystals using the molten metal flux method and examined them through single-crystal synchrotron X-ray diffraction, employing diamond anvil cells to exert pressures up to 20 GPa. Our analysis reveals insights into the anisotropic compressibility of these materials, highlighting the more compressible and flexible octahedral *A*-Bi bonds as the primary contributors to this anisotropy. Moreover, we observed a phase transition in both CaMg_2_Bi_2_ and YbMg_2_Bi_2_ at pressures above 9.6 and 8.7 GPa, respectively. The newly identified high-pressure phase exhibits a distortion of the original CaAl_2_Si_2_ structure with space group *C*2/*m*. This high-pressure structure is distinct from that of related compounds (e.g., CaMn_2_Bi_2_, MgMg_2_Bi_2_), the latter exhibiting a square pyramidal coordination for the *M* site. These results illustrate how pressure can reorganize bonding and coordination in *AM*_2_ *X*_2_ Zintl phases, providing insight into the structural mechanisms that govern phase stability. The anisotropy in compressibility may also influence directional phonon transport, with implications for tuning lattice thermal conductivity through pressure, strain, or chemical substitution.

## Graphical abstract

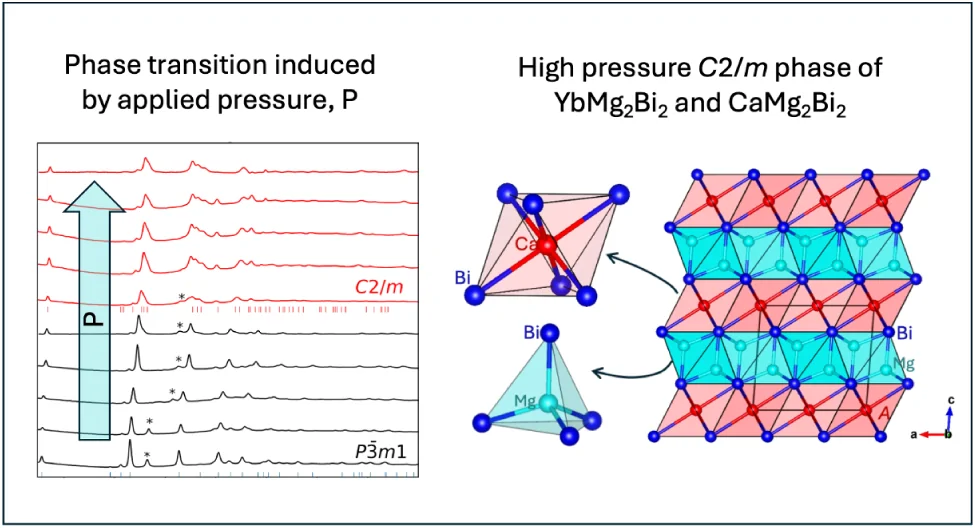

## Introduction

In recent years, compounds in the *AM*_2_ *X*_2_ stoichiometry that crystallize in the CaAl_2_Si_2_ structure type have been extensively studied for their thermoelectric performance^1–4^ quantum topological states^5,6^ and magnetic properties.^7,8^ Compounds that form the CaAl_2_Si_2_ structure type are highly tunable, as each *A*, *M*, and *X* site can accommodate a wide range of elements (*A* denotes rare-earth or alkaline-earth elements, *M* represents transition metals or group-13 elements, and *X* signifies group-14 or group-15 elements). The inherent tunability of this structure has inspired research efforts to explore the stability limits of this structure as well as the transport properties.^9^

For thermoelectric materials, low lattice thermal conductivity, κ_*L*_, is a necessary characteristic. In the limit of phonon transport limited by Umklapp scattering, κ_*L*_ ∝ *v* ^2^,^10^ where *v* is the velocity of collective lattice vibrations (*phonons*). It is often convenient to approximate the phonon velocity, *v*, as the low-frequency wave propagation speed, i.e., the *speed of sound*, *v*_*s*_, which in turn, is related to the bond stiffness, *k*, and mass of the atoms, *M* by $v_{s}∼\sqrt{k/M}$ .^11^ From these relations, it becomes evident that intrinsic low κ_*L*_ arises from soft bonds (low *k*) and/or high mass *M*. A number of prior studies^9,12–14^ have investigated bond stiffness in *A*Mg_2_Pn_2_ compounds via either acoustic measurements or via first-principles lattice dynamics calculations, showing that the *A*-site cation plays an important role. Comparisons of compounds with *A* = Mg versus *A* = Ca or Yb have shown that the larger cations (Ca and Yb) lead to higher experimental elastic constants and lower anharmonicity.^15^ However, only a few studies^16–18^ report their thermoelectric and topological properties, specifically for the CaMg_2_Bi_2_ and YbMg_2_Bi_2_ compounds. A less common approach to measuring bond stiffness is through the application of high pressure. By measuring the way in which the crystal structure evolves under applied pressure, one can simultaneously measure the compressibility of a solid and explore its stability limit with respect to high-pressure polymorphs.^19,20^ High pressure also can be used to tune electronic and thermal properties of a material, making it a valuable tool for investigating transport phenomena.^21^ In this context, high-pressure XRD experiments provide a connection between structural stability/bonding environments and lattice dynamics of these Zintl phases.

Although a large number of compounds form the CaAl_2_Si_2_ structure (space group *P*3̅*m*1), there have only been a handful of high-pressure crystallographic investigations. These initial studies hint at a surprising diversity of high-pressure structural responses:^22–25^ SrAl_2_Si_2_ and SrMn_2_P_2_ ^22,26^ transform into the well-known tetragonal (*I*4/*mmm*) crystal structure, while CaMn_2_Bi_2_ ^24^ and MgMg_2_(Sb,Bi)_2_ ^25^ were each reported to transform into a different monoclinic structure (with space group *P*2_1_/*m* and *C*2/*m*, respectively). The focus of the present work is on the two compounds, CaMg_2_Bi_2_ and YbMg_2_Bi_2_. Using in situ single crystal diffraction, we show that these compounds transform into a new monoclinic structure type. Further, we track the evolution of the crystal structure as a function of pressure to extract volumetric, linear, and bond-specific compressibility of the CaAl_2_Si_2_ structure. The present work aims to build on our prior work on MgMg_2_Bi_2_,^25^ by exploring how replacing Mg with larger Ca and Yb cations affects the stability of the ambient pressure phase and the details of the high pressure structure.

## Experimental Methods

### Polycrystalline Synthesis

For both the synthesis of single- and poly crystals, the following high-purity elements were used: Ca (dendritic pieces, 99.9%, Sigma-Aldrich), Mg (granules, Alfa Aesar 99.8%), Bi (shot, 99.99%, RotoMetal), and Yb (pieces, Sigma-Aldrich, 99.9%). Polycrystalline samples of CaMg_2_Bi_2_ and YbMg_2_Bi_2_ were synthesized by mixing stoichiometric amounts of their corresponding elements followed by spark plasma sintering (SPS). The elements were cut into small pieces and loaded into steel vials together with stainless-steel ball bearings inside an argon-filled glovebox. The airtight vials were then placed into a SPEX mill and ball-milled for 60 min. After milling, the vials were returned to the glovebox and the fine powder was loaded into graphite dies for SPS using a Dr. Sinter SPS-211LX.

The sintering procedure for both compounds was the same, where the powders were subjected to a pressure of 31 MPa, heated to 700 °C at a rate of 68 °C/min, and held at that temperature for 10 min. The target SPS temperature was selected to be 80% of the melting temperatures reported in ref 14, while the pressure and wait time were empirically determined to yield the desired phase. After that period, the pressure was immediately released and the samples were allowed to reach ambient temperature. The resulting consolidated disks were ground to fine powders and checked for phase purity using X-ray diffraction (XRD) with a Rigaku SmartLab Diffractometer with a Cu Kα source. The results of the Rietveld refinements, depicted in the SI Figure S1, revealed that the impurity content (elemental Bi) was less than 3% and 5% for CaMg_2_Bi_2_ and YbMg_2_Bi_2_, respectively.

### Single-Crystal Synthesis

Single crystals of CaMg_2_Bi_2_ and YbMg_2_Bi_2_ were grown via the self-flux method, with a starting composition of *A*Mg_4_Bi_6_ (*A* = Ca, Yb). No additional flux was added during the synthesis; the excess Mg and Bi relative to the *A*Mg_2_Bi_2_ target composition served as the self-flux. The high quality of the crystals was confirmed by sharp diffraction spots in the precession images, as demonstrated in Figure S2 (Supporting Information). After the ball-milling process, which was identical to the polycrystalline samples, the powders were then loaded into Canfield Al_2_O_3_ crucibles as part of crucible sets consisting of two alumina crucibles separated by an alumina frit disc and subsequently sealed in quartz ampules under vacuum (<10^–4^ Torr). The quartz ampules were then placed in a box furnace, where the furnace temperature was brought to 900 °C in 8 h, then to 850 °C in 1 h, and cooled to 650 °C in 40 h. After soaking for 48 h at 650 °C, the ampules were centrifuged to remove the liquid flux from the desired crystallized samples. During centrifugation, the liquid flux passed through the alumina frit disc into the second crucible, while the crystals were retained in the growth crucible. As a result, large crystals (approximately 5 mm in their longest dimension) were obtained. These crystals were then easily cleaved into smaller pieces with surgical blades, which yielded smaller pieces with plate-like geometry. The pieces that exhibited faceted morphology, and approximately 50 μm in their longest dimension, were selected and screened for crystallinity and phase purity using a Rigaku XtaLAB Synergy S Diffractometer.

### High-Pressure X-ray Diffraction

High-pressure diffraction experiments were conducted at the Advanced Photon Source (APS), Argonne National Laboratory, using diamond anvil cells and pressure-transmitting media to provide quasi-hydrostatic pressure conditions. For the experiments, either a 4:1 methanol–ethanol mixture or Ne was used as pressure-transmitting medium (this is specified below for each measurement). At room temperature, the 4:1 methanol–ethanol mixture remains hydrostatic to approximately 10 GPa, above which vitrification or solidification produces increasing deviatoric stress.^27,28^ Ne crystallizes near 4.8 GPa but remains highly quasi-hydrostatic in the solid state, with very low deviatoric stress reported to approximately 15 GPa.^28,29^ Accordingly, minor deviatoric stresses cannot be excluded, particularly at the highest pressures and near or above the hydrostatic limit of the methanol–ethanol medium. Small residual deviatoric stresses, together with the uncertainties inherent in high-pressure structural refinements, may produce isolated deviations in individual lattice parameters, bond lengths, or atomic coordinates. Because such deviations are generally not accompanied by correlated changes in other structural observables, they are not interpreted as evidence of additional phase transitions. X-ray diffraction patterns were collected during the compression cycle.

For powder diffraction of YbMg_2_Bi_2_, pairs of flat, 800-μm-culet diamond anvils (BX90 ^30^ design) were used. A precompressed powder of the desired composition was loaded into a 410-μm hole within a preindented steel gasket. A methanol–ethanol (4:1) mixture served as a pressure transmitting medium, and a 10-μm ruby crystal was used as the pressure indicator. The diffraction patterns were collected at beamline 13-BM-C, using X-rays with a wavelength λ = 0.413 Å (30 keV). CaMg_2_Bi_2_ powder diffraction experiments were carried out at APS beamline 16 BM-D, with X-ray light of λ = 0.434 Å (28.6 keV), using flat, 300-μm-culet diamond anvils (BX90 design), and neon as the pressure transmitting medium, using the COMPRES-GSECARS gas loading system.^31^ A preindented rhenium gasket was used, in which a hole of ∼150 μm in diameter was drilled.

For single crystal experiments, diamond anvil pairs of flat 300-μm culets (custom CVD, conical Boehler-Almax design, with (Vascomax) stainless steel were used, with an effective aperture of 80°. Prescreened single crystals of the desired compositions were loaded into a 150-μm hole within a preindented Re gasket. A 4:1 methanol–ethanol mixture was used as the pressure-transmitting medium for CaMg_2_Bi_2_, whereas Ne was used for YbMg_2_Bi_2_. A 10-μm ruby crystal was used as the pressure indicator. Ne gas was loaded at GSECARS (Sector 13)^31^ at APS. Single-crystal X-ray diffraction (SC-XRD) experiments were carried out in beamline 13-BM-C, with X-ray wavelength source of λ = 0.434 Å (28.6 keV). The diffraction patterns were collected with a Dectris Pilatus3 1 M Pixel Array Detector.

The diffraction data for single-crystalline CaMg_2_Bi_2_ and YbMg_2_Bi_2_ were collected by APEX3,^32^ integrated using SAINT^33^ code and corrected for absorption with the SADABS method.^34^ The structure was solved by the SHELXS routine.^35^ For both compositions, the structures were refined in the OLEX2 Suite,^36^ by the full-matrix least-squares methods of SHELXL. All atomic coordinates and bond-specific structural parameters were obtained from the single-crystal data. The powder data were used to determine the phase-transition ranges, supplement the equation-of-state analysis, and verify the high-pressure structural model through Rietveld refinement (see SI Figure S3). Rietveld refinements were performed in GSAS-II.^37^

After the crystallographic refinements, the parameters of the equations of state (EoS) for the two compounds were fit to data via the least-squares fitting routine of the EoSFit7 software.^38^ The volume compressibilities were fit using a second-order Birch–Murnaghan (BM) EoS.^39,40^ Polyhedral volumes and uncertainties were calculated from bond lengths using the approach described in the Supporting Information Section S4.

### Safety Statement

No uncommon hazards are noted.

## Results and Discussion

The powder and single crystals of CaMg_2_Bi_2_ and YbMg_2_Bi_2_ form the trigonal CaAl_2_Si_2_ structure (space group *P*3̅*m*1), as reported in prior literature.^41,42^ The powder XRD patterns on polycrystalline samples were initially collected for CaMg_2_Bi_2_ and YbMg_2_Bi_2_ as a function of pressure up to 18 and 17.5 GPa, respectively. Figure 1 shows the patterns at selected pressures. In both samples, the trigonal phase is shown in black. New peaks begin to appear in the diffraction patterns of CaMg_2_Bi_2_ and YbMg_2_Bi_2_ above 9.6 and 8.7 GPa, respectively, indicating the beginning of a phase transition.

**Figure 1:**
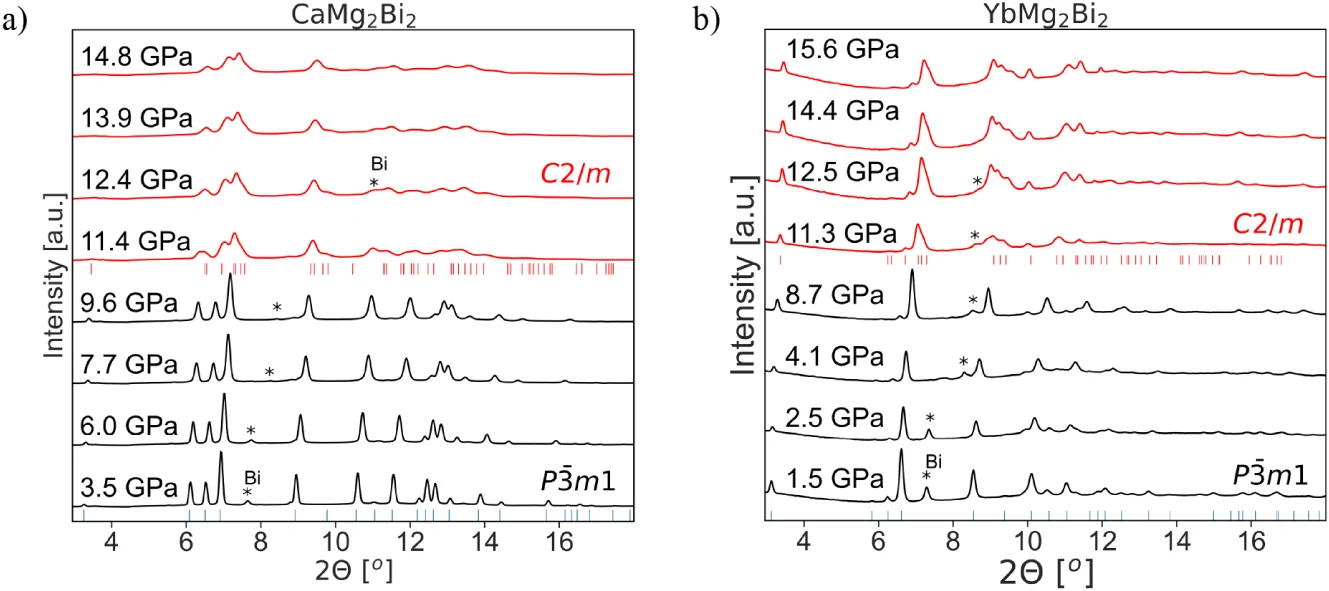
Powder XRD for a) CaMg_2_Bi_2_ and b) YbMg_2_Bi_2_ as a function of pressure shows evidence of phase transitions in the range of 9.6–11.4 GPa and 8.7–11.3 GPa, respectively. The trigonal phase (*P*3̅*m*1) is shown in black, and the high pressure monoclinic (*C*2/*m*) phase is shown in red. Elemental Bi is present as an impurity in both samples, marked by asterisks.

Rietveld analysis of the powder diffraction data above 11.3 GPa was initially attempted using two known high-pressure structures of similar compounds, namely that of *C*2/*m*-Mg_3_Sb_2_ ^25^ and *P*2_1_/*m*-CaMn_2_Bi_2_.^24^ However, these solutions did not provide an acceptable fit. For that reason, single-crystal XRD experiments were conducted for both compositions, ultimately showing that CaMg_2_Bi_2_ and YbMg_2_Bi_2_ crystallize in a new structure type with space group *C*2/*m*. In the following sections, we present first an analysis of the compressibility of each compound within the stability range of the trigonal phase, followed by a description of the high-pressure structures of both compounds, based on the single-crystal data. Tables S1 and S2 in the Supporting Information summarize selected single-crystal crystallographic data for the trigonal phases of CaMg_2_Bi_2_ and YbMg_2_Bi_2_, respectively, relevant to the Birch–Murnaghan equation-of-state analysis. Table S3 summarizes the crystallographic data for the high-pressure monoclinic *C*2/*m* phases, while Tables S4 and S5 report the corresponding refined atomic parameters for CaMg_2_Bi_2_ and YbMg_2_Bi_2_, respectively.

### Compressibility of CaMg_2_Bi_2_ and YbMg_2_Bi_2_

#### Volume Compressibility

The experimental unit cell volume as a function of pressure is shown in Figure 2a–b for CaMg_2_Bi_2_ and YbMg_2_Bi_2_.

**Figure 2:**
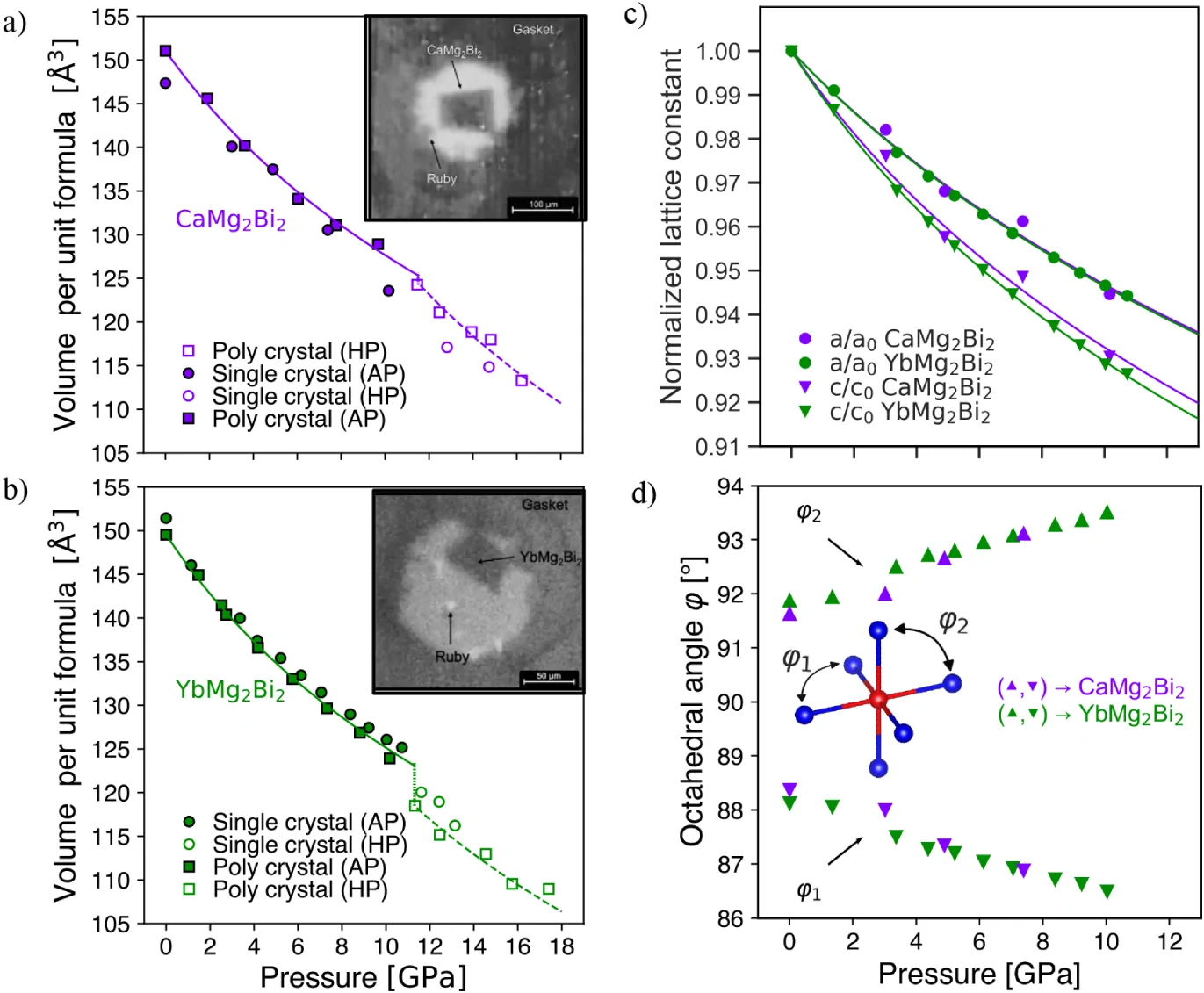
Experimental single-crystal (circles) and polycrystalline (squares) unit cell volumes as a function of pressure for a) CaMg_2_Bi_2_ and b) YbMg_2_Bi_2_ and their corresponding images inside DACs after pressure-medium loading (see insets, methanol–ethanol for CaMg_2_Bi_2_ and Ne for YbMg_2_Bi_2_). The open symbols in a) and b) show the volume/formula unit of the high-pressure phase. (c) Normalized lattice parameters and (d) octahedral angles vs pressure for CaMg_2_Bi_2_ (magenta) and YbMg_2_Bi_2_ (green) from SC-XRD. Solid curves in panels (a) and (b) represent second-order Birch–Murnaghan fits to the ambient-pressure phases. Dashed curves through the high-pressure data are guides to the eye used only to estimate the apparent volume discontinuities. Solid lines in panel (c) represent the linearized second-order Birch–Murnaghan fits.

Filled symbols correspond to the ambient-pressure trigonal structure type and open symbols to the high-pressure structure type, which will be discussed further in the next section. The volumetric bulk modulus for the trigonal ambient-pressure (*B*_0_) phases were calculated by fitting the pressure–volume data (using both single and polycrystalline data points) to the second-order Birch–Murnaghan (BM) equation of state,^39,40,43^

1 $$P\left(V\right)=\frac{3B_{0}}{2}\left[{\left(\frac{V_{0}}{V}\right)}^{7/3}-{\left(\frac{V_{0}}{V}\right)}^{5/3}\right]$$

where *P* is the applied pressure (determined from the ruby standard), *V* the observed unit cell volume, and *V*_0_ the reference unit cell volume. In the second-order BM fit, *B*_0_ is the fitted zero-pressure modulus and $B_{0}^{\prime }$ is fixed at 4. The second order BM fits to the volume data are shown as the purple and green curves in Figure 2, and the resulting *B*_0_ values are listed in Table 1. A third-order Birch–Murnaghan equation of state was also considered. Although the *F*–*f* analysis presented in Figure S4 suggests a value of $B_{0}^{\prime }$ below 4, unconstrained third-order fits produce strongly correlated and poorly determined values of *B*_0_ and $B_{0}^{\prime }$ . The second-order fits were therefore retained because they provide stable values of *B*_0_ that agree closely with independent ambient-pressure acoustic measurements.^15^ CaMg_2_Bi_2_ and YbMg_2_Bi_2_ were found to have nearly identical zero-pressure *B*_0_ (∼37–38 GPa). This is not surprising, given the similar ionic radii of Yb^2+^ (1.02 Å^44^) and Ca^2+^ (1.00 Å^44^), and similar ambient pressure volume. Our results are also in agreement with previously reported values obtained using ultrasonic measurements on bulk polycrystalline samples (36 and 38 GPa for CaMg_2_Bi_2_ and YbMg_2_Bi_2_, respectively.^15^

**Table 1:** Summary of the Fitted Ambient-Pressure Moduli for the Trigonal CaMg_2_Bi_2_ and YbMg_2_Bi_2_ Compounds^a^

Zero-pressure moduli (GPa)	CaMg_2_Bi_2_	YbMg_2_Bi_2_
Bulk modulus (AP), *B*_0_	37 ± 3	37.6 ± 0.4
Linear modulus along a, *M*_*a*_	131.9 ± 5	130.5 ± 3
Linear modulus along c, *M*_*c*_	94.1 ± 3	87.8 ± 0.8
Tetrahedron modulus, *K*_*tet*_	48 ± 1	42.4 ± 0.25
Octahedron modulus, *K*_*oct*_	27.3 ± 0.15	30.4 ± 0.1
Modulus of bond *d*1, *K*_*d*1_	–	96 ± 1.5
Modulus of bond *d*2, *K*_*d*2_	–	168 ± 15
Modulus of bond *d*3, *K*_*d*3_	–	119 ± 2.5

a Bulk modulus (*B*_0_), linear moduli in *a*- and *c*-directions (*M*), and polyhedral moduli (*K*_*tet*_, *K*_*oct*_), and bond-specific moduli (*K*_*d*1_, *K*_*d*2_, *K*_*d*3_) are included.

At the transition from the trigonal to monoclinic structure, extrapolation of the low- and high-pressure volume trends suggests apparent decreases in the volume per formula unit of ∼0.9% for CaMg_2_Bi_2_ and ∼3.7% for YbMg_2_Bi_2_. Although the nominal volume decrease is larger for YbMg_2_Bi_2_, these values should be interpreted cautiously. The high-pressure phases are represented by relatively few data points, with possible phase coexistence over finite pressure intervals. The present data therefore suggest modest apparent volume discontinuities, but we are not confident that the difference in magnitude is intrinsic. Above the phase transition, we have insufficient data points to reliably determine the elastic moduli of the high-pressure structure type (the lattice parameters vs pressure for the *C*2/*m* are shown in the Figure S5). However, BM fits are shown as dashed curves to provide a ″guide to the eye″, and these were to estimate the change in volume at the phase transition pressure.

#### Linear Compressibility

Returning to the trigonal structure type, Figure 2c shows the normalized lattice parameters, *a*/*a*_0_ and *c*/*c*_0_, as a function of pressure, obtained from single crystal data. From these, the linear bulk moduli (*M*_*a*_ and *M*_*c*_) were extracted using a linearized 2nd order BM equation, given by

2 $$P\left(l\right)=\frac{3M_{l}}{2}\left[{\left(\frac{l_{0}^{3}}{l^{3}}\right)}^{7/3}-{\left(\frac{l_{0}^{3}}{l^{3}}\right)}^{5/3}\right]$$

Here, *l* represents either the lattice parameter *a* or *c*.^45^ The values at ambient pressure, *a*_0_ and *c*_0_, were fixed to the independently measured ambient-pressure values. Figure 2c clearly shows that *c* is more compressible than *a* for both compounds. The corresponding evolution of the octahedral angles, ϕ_1_ and ϕ_2_, with pressure is shown in Figure 2d and is discussed below in relation to the polyhedral compressibility. Accordingly, the linear moduli obtained from the BM fits along *c* are much smaller (*M*_*c*_ ∼ 88–94 GPa) than those obtained along *a* (*M*_*a*_ ∼ 130–132 GPa). Figure 3 shows a comparison of the bulk and linear elastic moduli of MgMg_2_Bi_2_, CaMg_2_Bi_2_, and YbMg_2_Bi_2_.

**Figure 3:**
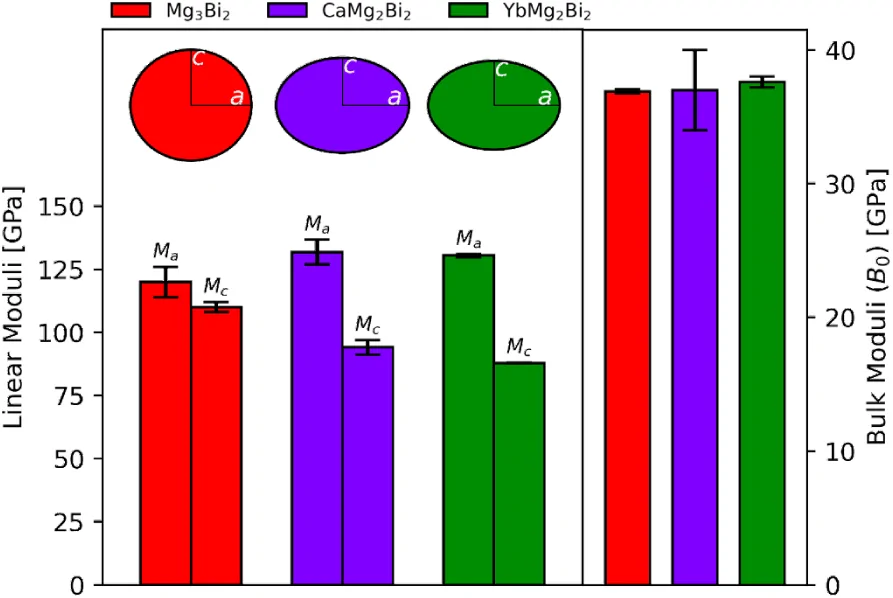
Linear (left) and bulk (right) moduli comparison for *A*Mg_2_Bi_2_ (*A* = Ca, Mg, Yb) at ambient pressure with their corresponding uncertainties. MgMg_2_Bi_2_ data taken from ref 25.

The ellipses illustrate how the linear modulus varies as a function of crystallographic direction in the a–c plane. Conceptually, they can be understood as the final cross sections of spherical particles subjected to isostatic pressure. We can see that even though the bulk moduli are nearly identical regardless of cation species, the degree of anisotropy varies strongly. Compared with MgMg_2_Bi_2_, the larger cations in the present compounds lead to decreased stiffness (smaller *M*_*c*_) along the *c*-axis, and increased stiffness along the a- and b-axes (larger *M*_*a*_). Such anisotropic compressibility can also give rise to anisotropic lattice thermal conductivity, as demonstrated by Zhang et al., who linked directional differences in bonding and stiffness in CaAl_2_Si_2_-type compounds to corresponding anisotropy in phonon velocities, anharmonicity, and κ_*L*_.^46^

#### Compressibility of Polyhedra and Individual Bonds

To better understand the underlying factors controlling the anisotropic compressibility, we consider how individual bond lengths and bond angles evolve as a function of pressure.^47^ As shown in Figure 4b, the trigonal *A*Mg_2_Bi_2_ (*A* = Ca, Yb) structure consists of layers of [Mg_2_Bi_2_]^2–^, in which the Mg atom (cyan) is tetrahedrally coordinated by Bi (blue).

**Figure 4:**
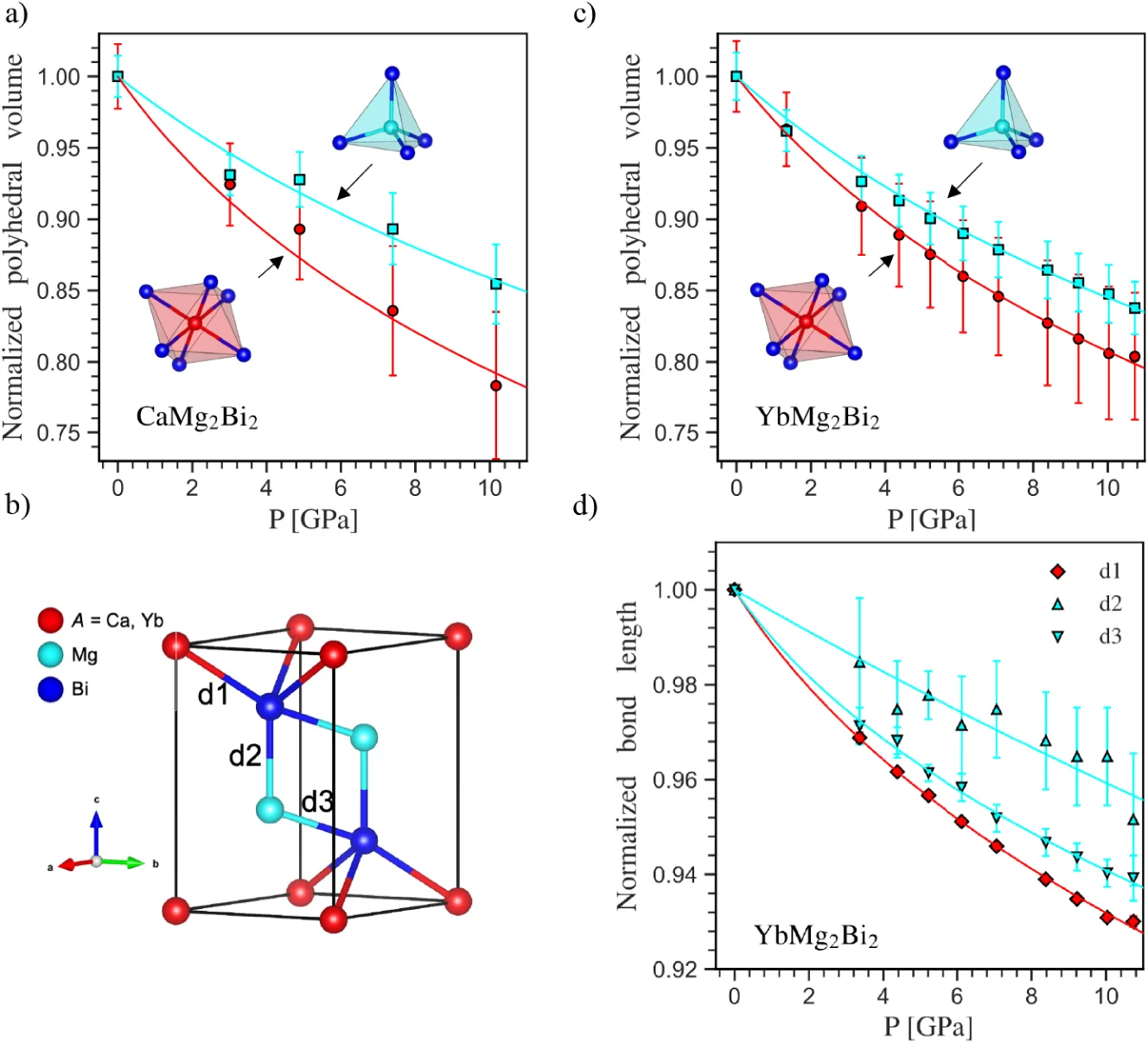
(a) Normalized polyhedral volume vs pressure below the phase transition for CaMg_2_Bi_2_ from SC-XRD. (b) Ambient-pressure trigonal structure of *A*Mg_2_Bi_2_ (*A* = Ca, Yb), space group *P*3̅*m*1, showing the octahedral (*d*1) and tetrahedral bonds (*d*2 and *d*3). (c) Normalized polyhedral volume vs pressure below the phase transition for YbMg_2_Bi_2_ from SC-XRD. (d) Normalized bond length vs pressure for YbMg_2_Bi_2_ from SC-XRD, highlighting the greater compressibility of the octahedral bond.

Each tetrahedron shares three edges with neighboring tetrahedra. The tetrahedral layers are separated by the divalent cations (red), i.e., trigonal layers of cations (Ca^2+^ or Yb^2+^) that are octahedrally coordinated by six Bi atoms. The *A*-centered octahedra possess one crystallographically unique bond length (*d*1). In contrast, the tetrahedra have two distinct bonds, one apical bond (*d*2) and three basal bonds (*d*3). Accurate Mg positions, which are needed to obtain *d*2 and *d*3 as a function of pressure, could only be refined from the YbMg_2_Bi_2_ SC-XRD data, but not for CaMg_2_Bi_2_. Therefore, the pressure-dependent bond lengths are only shown for YbMg_2_Bi_2_ (Figure 4d). However, the overall volume of each polyhedral environment (octahedral and tetrahedral) as well as the Bi-*A*-Bi bond angles could be obtained using only the refined Bi position, and was therefore calculated for both compounds.

The polyhedral volumes as a function of pressure are shown in Figure 4a and c. The uncertainty was estimated using an error propagation method discussed in detail in the Supporting Information Section S4. The experimental data suggest that for both compounds, but most clearly in the case of YbMg_2_Bi_2_, the octahedral environment compresses at a faster rate than the tetrahedral environment. This can be quantified using a modified version of eq 1, in which the unit cell volume is replaced by the polyhedral volume, yielding zero-pressure “polyhedra bulk moduli” (defined here as *K*_*oct*_ and *K*_*tet*_, listed in Table 1.^48^ The BM fits are shown as solid curves in Figure 4a and c. For both CaMg_2_Bi_2_ and YbMg_2_Bi_2_, the tetrahedron bulk modulus, *K*_*tet*_, is considerably larger than the octahedron bulk *K*_*oct*_. As a result, in both compounds, the overall bulk modulus lies between that of the tetrahedral modulus and the octahedral modulus.

Diving deeper, we find that the octahedral environment is more compressible than the tetrahedral one for two reasons; first, the octahedral bonds are more compliant, and second, the octahedral angles vary with pressure, allowing for further volume compression. The former can be seen in Figure 4d, which shows *d*1, *d*2, and *d*3 as a function of pressure in YbMg_2_Bi_2_. Note that uncertainty in the positions of the light Mg atoms leads to large uncertainty in *d*2 and *d*3. Initial values of bond lengths for YbMg_2_Bi_2_ are *d*1 = 3.2838(3) Å, *d*2 = 2.8990(3) Å, *d*3 = 2.9689(2) Å; Figure 4d clearly shows a faster compression with pressure of the octahedral bond *d*1 with respect to the other bonds. The individual bond moduli (*K*_*di*_, where i = 1, 2, or 3) were obtained using a modified version of eq 2, and are reported in Table 1. The 2nd-order BM fits are shown as dashed lines in Figure 4d. The octahedral bond angles, ϕ_1_ and ϕ_2_, are also allowed to vary in the trigonal symmetry. As shown in Figure 2d, these two angles evolve with pressure in CaMg_2_Bi_2_ and YbMg_2_Bi_2_. In an ideal octahedron, these angles would be 90°. At ambient pressure, the angles are 88° and 92° (they are nearly identical regardless of cation species). As pressure increases, these angles deviate further from 90°, indicating a distortion of the ideal octahedral geometry, allowing the structure to further compress in the *c*-direction. This flexibility, together with the more compliant octahedral bonds, explains why the *c*-axis is more compressible (softer) than the *a*-axis in this structure type.

### High-Pressure Crystal Structure

As previously mentioned, a high-pressure structural transformation for both compounds was first observed in the powder samples at pressures between 9.6 and 11.4 GPa and 8.7–11.3 GPa in CaMg_2_Bi_2_ and YbMg_2_Bi_2_, respectively. The single crystal data confirmed these phase transition pressures. Although the use of complementary powder and single-crystal diffraction measurements with different pressure-transmitting media supports an intrinsic origin for the structural transitions observed in this study, residual deviatoric stresses may contribute to the scatter in individual data points as well as to uncertainty in the precise transition pressures.

The high-pressure structures were refined as having the monoclinic crystal system with a unit cell corresponding to twice that of the *AM*_2_ *X*_2_ unit cell. The Bi and *A* = Ca, Yb positions were stable and consistent across multiple models tested during the refinement. However, refinement of the Mg positions from the single-crystal data for both compounds proved to be challenging because of the relatively weak X-ray scattering from Mg compared with Bi and the *A*-site cations. Several possible solutions with similar R values were explored, each of which had nonphysically short Mg–Mg distances within the Mg–Bi layers. Ultimately, for both compounds, the only solutions with physical interatomic distances are those with the Mg atoms located in a tetrahedral coordination with Bi, representing only a slight distortion of the ambient-pressure structure. The final refined space group was *C*2/*m* (Table S3), and the atomic positions are given in Tables S4 and S5. After refining the structure using single-crystal data, we cross-checked the solution against polycrystalline data using Rietveld refinements, which are shown in Figure S3. Despite pressure-induced peak broadening in the polycrystalline data, the high-pressure structural model remains robust and was successfully verified for both compounds.

The high-pressure (*C*2/*m*) structure, shown in Figure 5a, is best described as a monoclinically distorted derivative of the ambient-pressure CaAl_2_Si_2_-type structure. The transition removes the 3-fold rotational symmetry of the *P*3̅*m*1 phase and introduces monoclinic shear, with β = 94.36(4)° for CaMg_2_Bi_2_ and β = 91.82(7)° for YbMg_2_Bi_2_. Correspondingly, the site symmetry of the *A* cation decreases from (3̅*m*) to (2/*m*), while those of Mg and Bi decrease from (3*m*) to (*m*). However, the refined Mg and Bi coordinates remain close to the positions expected for the parent trigonal structure, indicating that the transformation involves relatively small atomic displacements coupled with anisotropic lattice strain and shear. The *A*Bi_6_ octahedra and MgBi_4_ tetrahedra remain intact, but the loss of 3-fold symmetry splits previously equivalent bond lengths and angles, producing more distorted coordination environments. Thus, the high-pressure phase retains the connectivity and principal coordination motifs of the ambient-pressure structure while accommodating compression through bond-length splitting, polyhedral distortion, and relative displacement of the layers.

**Figure 5:**
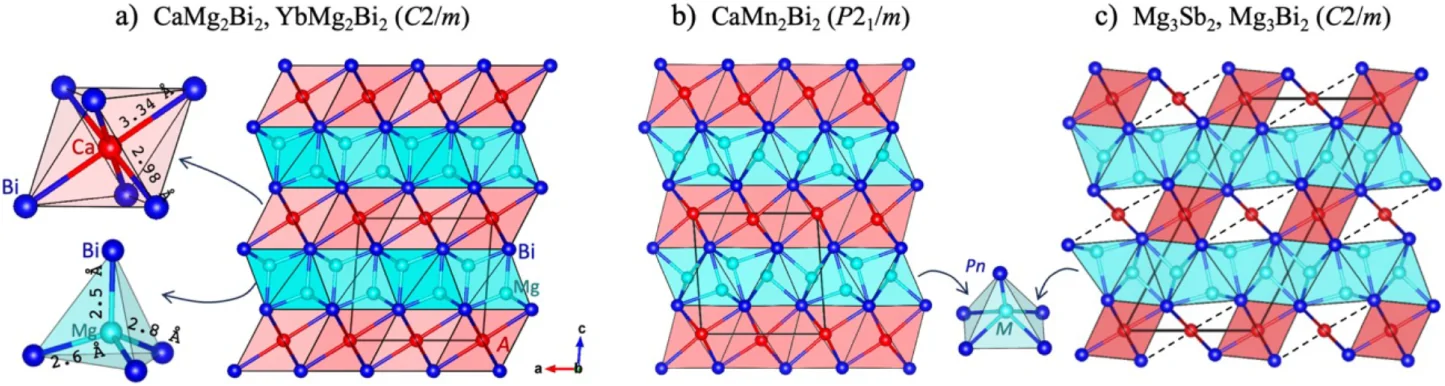
a) High-pressure (*C*2/*m*) structures for CaMg_2_Bi_2_ and YbMg_2_Bi_2_ with detailed octahedron (top left) and detailed tetrahedron (bottom left). Bond lengths are given for CaMg_2_Bi_2_ at 12.82 GPa. In the high-pressure structures of b) CaMn_2_Bi_2_ space group *P*2_1_/*m* (structural data taken from ref 24), and c) MgMg_2_Pn_2_ (*Pn* = Sb, Bi), space group *C*2/*m*, the ambient-pressure tetrahedral environment becomes square pyramidal (structural data from ref 25).

As shown in Figure 5b–c, other *AM*_2_ *X*_2_ compounds, namely MgMg_2_Pn_2_ (*Pn* = Sb, Bi) and CaMn_2_Bi_2_, also undergo displacive transformations from trigonal to monoclinic symmetry under pressure, but with more substantial changes in coordination than those observed here. In CaMn_2_Bi_2_, a monoclinic *P*2_1_/*m* phase with *Z* = 2 forms above 2 GPa;^24^ the tetrahedral MnBi_4_ units transform into square-pyramidal coordination environments, shown by the cyan polyhedra in Figure 5b, while the Ca-centered octahedra undergo only modest bond elongation. In MgMg_2_Bi_2_ and MgMg_2_Sb_2_, monoclinic *C*2/*m* phases with *Z* = 4 form above 7.8 and 4.0 GPa, respectively.^25^ These transitions likewise convert the tetrahedral Mg–*Pn* environments into square pyramids, but are additionally accompanied by the breaking of octahedral bonds at alternating *A*-cation sites, as indicated by the dashed lines in Figure 5c. By contrast, the high-pressure phases of CaMg_2_Bi_2_ and YbMg_2_Bi_2_ retain both the MgBi_4_ tetrahedra and the *A*Bi_6_ octahedra, with compression accommodated primarily through bond-length splitting and polyhedral distortion. The particularly extensive disruption of the *A*-site octahedra in the *A* = Mg compounds may reflect the small size of Mg in this coordination environment. Within *A*Mg_2_ *X*_2_ compounds, replacing *A*-site Mg with the larger Ca or Yb cations may better stabilize the parent octahedral framework, contributing to higher transition pressures and high-pressure structures that more closely preserve the ambient-pressure coordination topology.

## Conclusion

Single crystal and powder diffraction at high pressure reveal that CaMg_2_Bi_2_ and YbMg_2_Bi_2_ exhibit strongly anisotropic compression and undergo closely related pressure-induced structural transitions. Their greater compressibility along the *c*-direction arises primarily from the compliance of the *A*–Bi bonds within the *A*Bi_6_ octahedra, whereas the comparatively rigid MgBi_4_ tetrahedra limit compression in the *a*-direction. Above 9.6 GPa for CaMg_2_Bi_2_ and 8.7 GPa for YbMg_2_Bi_2_, the ambient-pressure trigonal *P*3̅*m*1 phase undergoes a transformation to a monoclinic *C*2/*m* phase, via a simple displacive distortion. In the *C*2/*m* phase, the alternating layers of *A*-centered octahedral and Mg-centered tetrahedral motifs are retained, with compression accommodated through anisotropic bond contraction, bond-angle changes, and mild polyhedral distortion. This is in contrast to previously reported high-pressure modifications of CaAl_2_Si_2_-type materials, which show substantial changes to coordination environments of both sites. Overall, when combined with our prior work on Mg_3_Bi_2_ and Mg_3_Sb_2_, the present study demonstrates that the larger cations (Ca and Yb in this case) both amplify the anisotropic compressibility of the trigonal phase and stabilize the parent coordination framework under pressure, favoring transformations dominated by subtle polyhedral distortions rather than extensive coordination rearrangements.

## Supplementary Material



## Notes

Crystallographic data for the ambient- and high-pressure phases, Rietveld refinements, *F*–*f* analysis, equation-of-state results, polyhedral-volume calculations, and uncertainty estimates (PDF)

## #

Mechanical and Aerospace Engineering, The George Washington University, Washington, DC. 20052, USA
