# Supplementary material for: Compressibility and High-Pressure Structure of CaMg2Bi2 and YbMg2Bi2
Source: Inorg Chem. 2026 Sep 22;65(39):22996–3004. doi: 10.1021/acs.inorgchem.6c02769 (PMC13644305; doi:10.1021/acs.inorgchem.6c02769)
Supplement: Supplementary file 1 [file ic6c02769_si_001.pdf]

Supplemental Information - Compressibility and High-Pressure Structure of $CaMg_2Bi_2$ and $YbMg_2Bi_2$

Mario Calderón-Cueva[1,a], Allison Pease[2], Cheng Peng[3], Wanyue Peng[1], Megan Rylko[1], Weiwei Xie[3], Susannah M. Dorfman[2], and Alexandra Zevalkink[1,*]

[1]Chemical Engineering and Materials Science Department, Michigan State University, East Lansing, MI 48824, USA
[2]Department of Earth and Environmental Sciences, Michigan State University, East Lansing, MI 48824, USA
[3]Chemistry Department, Michigan State University, East Lansing, MI 48824, USA
[*]E-mail: alexzev@msu.edu
[a]Current Address: Mechanical and Aerospace Engineering, The George Washington University, Washington, D.C. 20052, USA

S1 Rietveld refinements at ambient pressure

(a)

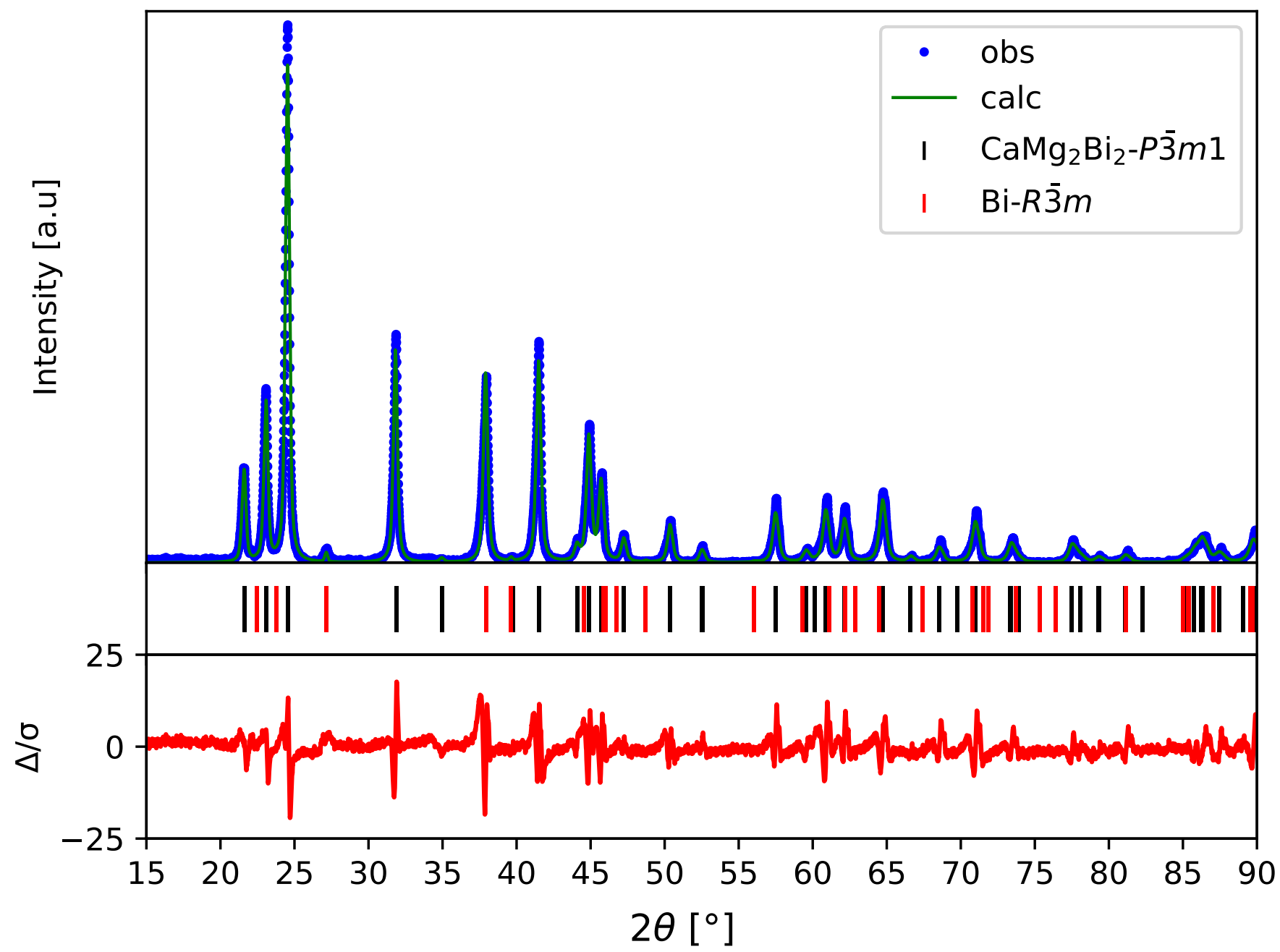

(b)

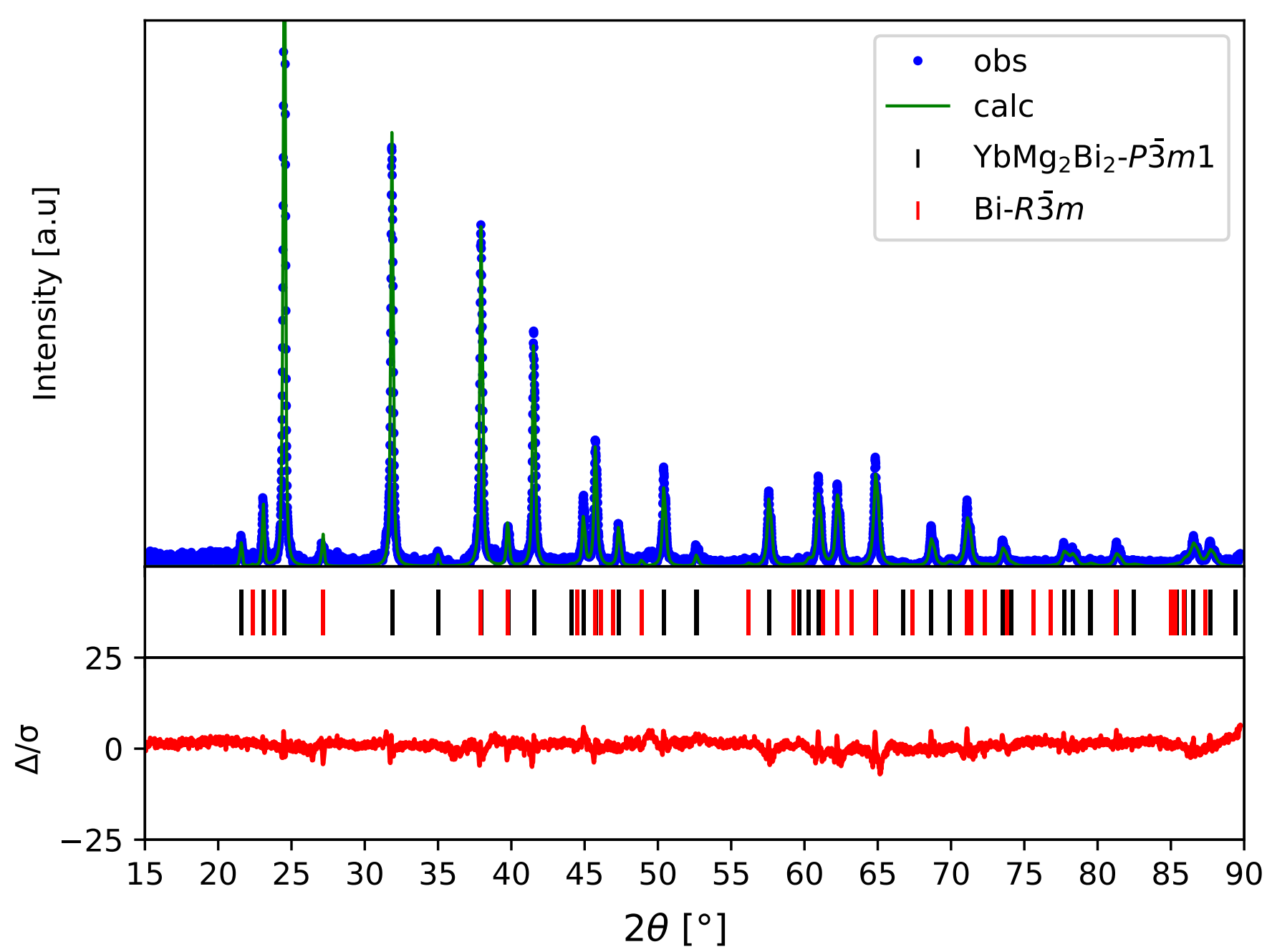

Figure S1: Ambient-pressure ($P\bar{3}m1$) Rietveld refinements of a) $CaMg_2Bi_2$ and b) $YbMg_2Bi_2$ collected at 1 atm. In a), the Bi impurity phase is around 3%. In b), the content of Bi is approx. 5%.

S2 Selected crystallographic data for the ambient- and high-pressure phases

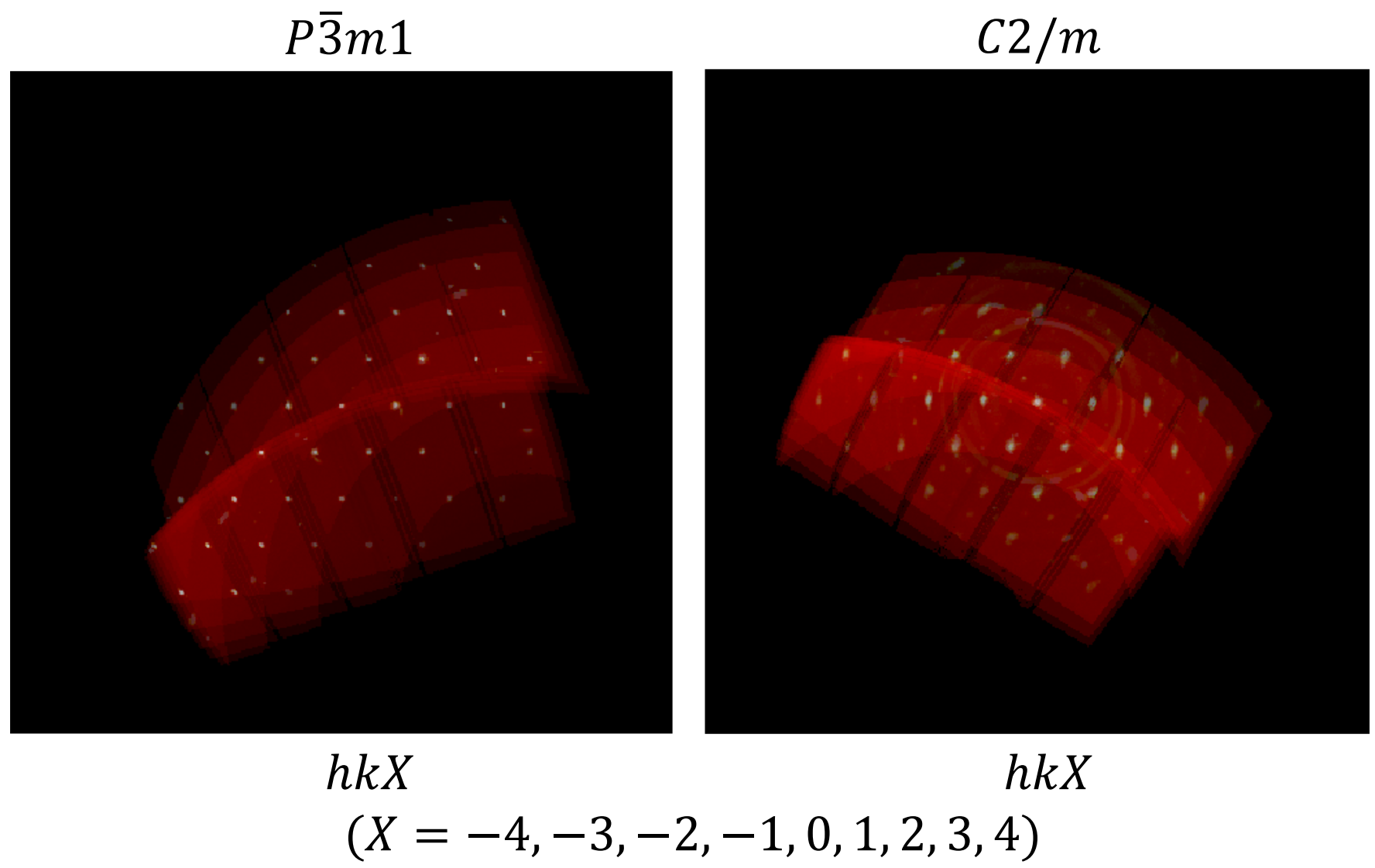

Figure S2: Precession images for $YbMg_2Bi_2$ at pressures below (left) and above (right) the transition. The crystal quality below the transition pressure is excellent, while significant broadening of reflections is observed above the transition pressure. However, there is clear evidence of decreased symmetry, consistent with the powder diffraction patterns.

Table S1: Crystallographic data for the trigonal $CaMg_2Bi_2$ structure at different pressures from single crystal XRD experiments.

Pressure (GPa)	AP	3.0	4.9	7.4	10.2
Space Group; Z	$P\bar{3}m1$; 1	$P\bar{3}m1$; 1	$P\bar{3}m1$; 1	$P\bar{3}m1$; 1	$P\bar{3}m1$; 1
a (Å)	4.71886(17)	4.651(2)	4.6223(15)	4.549(2)	4.472(2)
b (Å)	4.71886(17)	4.651(2)	4.6223(15)	4.549(2)	4.472(2)
c (Å)	7.6412(3)	7.472(4)	7.431(3)	7.283(4)	7.135(4)
Volume (Å^3)	147.356(10)	139.97(16)	137.49(8)	130.54(10)	123.57(11)
Meas. Reflections; R_{int}	313; 12.98	109; 5.78	121; 5.96	144; 7.76	131; 5.18
No. of indep. reflections	28	56	71	119	80
R_1; wR_2 (all intensities)	10.88; 23.57	12.29; 33.32	17.38; 44.00	12.59; 30.12	14.99; 48.20
GoF	1.4147	1.8207	1.4868	1.5871	2.7204

Table S2: Crystallographic data for the trigonal $YbMg_2Bi_2$ structure at different pressures from single crystal XRD experiments.

Pressure (GPa)	AP	3.4	5.2	7.1	10.0
Space Group; Z	$P\bar{3}m1$; 1	$P\bar{3}m1$; 1	$P\bar{3}m1$; 1	$P\bar{3}m1$; 1	$P\bar{3}m1$; 1
a (Å)	4.7149(4)	4.6502(11)	4.6034(4)	4.5624(6)	4.5058(8)
b (Å)	4.7149(4)	4.6502(11)	4.6034(4)	4.5624(6)	4.5058(8)
c (Å)	7.6063(7)	7.474(3)	7.3782(7)	7.2937(10)	7.1702(13)
Volume (Å^3)	146.43(2)	139.98(7)	135.406(19)	131.48(3)	126.07(4)
Meas. Reflections; R_{int}	152; 6.66	65; 2.42	126; 3.18	123; 2.80	126; 3.08
No. of indep. reflections	1096	161	347	324	315
R_1; wR_2 (all intensities)	2.99; 7.05	3.83; 11.58	4.94; 27.52	5.71; 18.17	7.09; 20.31
GoF	1.106	1.369	1.422	1.228	1.286

Table S3: Crystallographic data for the monoclinic phase of $CaMg_2Bi_2$ and $YbMg_2Bi_2$.

	$CaMg_2Bi_2$	$YbMg_2Bi_2$
Pressure (GPa)	12.8	11.6
Space Group; Z	$C2/m$; 2	$C2/m$; 2
a (Å)	7.65(3)	7.558(7)
b (Å)	4.333(3)	4.489(6)
c (Å)	7.082(10)	7.081(6)
β (°)	94.36(4)	91.82(7)
Volume (Å^3)	234.2(9)	240.1(4)
Meas. Reflections; R_{int}	70; 1.54	128; 3.90
No. of indep. Reflections	69	290
R_1; wR_2 (all intensities)	9.60; 26.20	18.31; 50.16
GoF	1.525	2.043

Table S4: Structure parameters of the high-pressure structure for $CaMg_2Bi_2$ at 12.8 GPa. Note that the uncertainties for the Mg atom are significantly higher than those observed for Ca and Bi. These elevated values strongly suggest significant disorder or positional uncertainty for the Mg atom.

Atom	x	y	z	Occ.	U_{eq} (Å^2)	Site	Sym.
Ca	1.000(3)	1.000(3)	0.000(1)	1.000	0.020(12)	2a	2/m
Bi	0.648(2)	1.000(3)	0.253(1)	1.000	0.028(9)	4i	m
Mg	0.850(17)	0.500(30)	0.385(11)	1.000	0.030*	4i	m

*The Mg isotropic displacement parameter was not refined; therefore, no uncertainty is reported for U_{eq} of Mg.

Table S5: Structure parameters of the high-pressure structure for $YbMg_2Bi_2$ at 11.6 GPa.

Atom	x	y	z	Occ.	U_{eq} (Å^2)	Site	Sym.
Yb	0.500(1)	0.500(1)	1.000(1)	1.000	0.050(4)	2a	2/m
Mg	0.161(6)	0.500(1)	0.647(4)	1.000	0.037(7)	4i	m
Bi	0.172(1)	0.500(1)	0.239(1)	1.000	0.050(3)	4i	m

S3 Rietveld refinements at high pressure

(a)

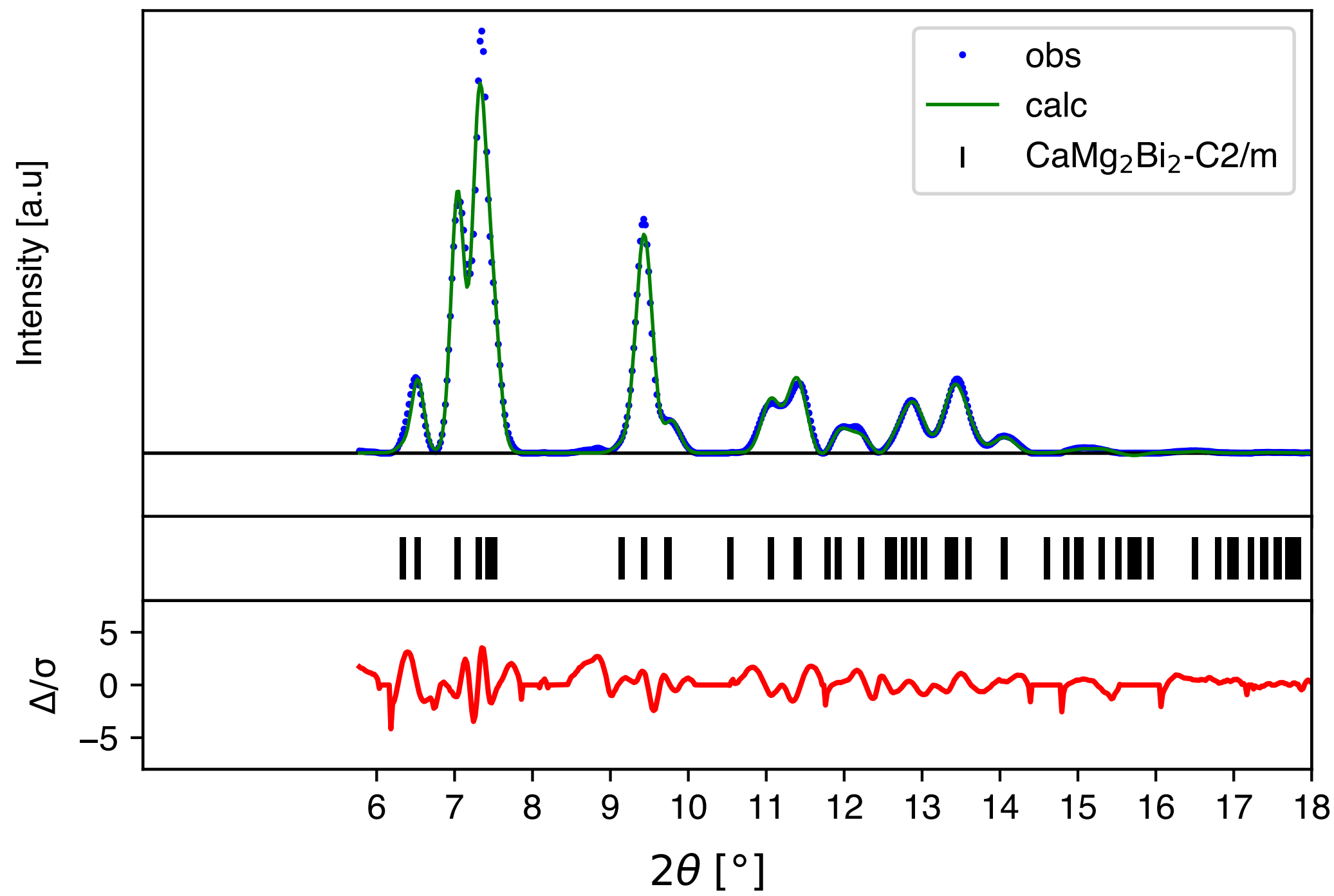

(b)

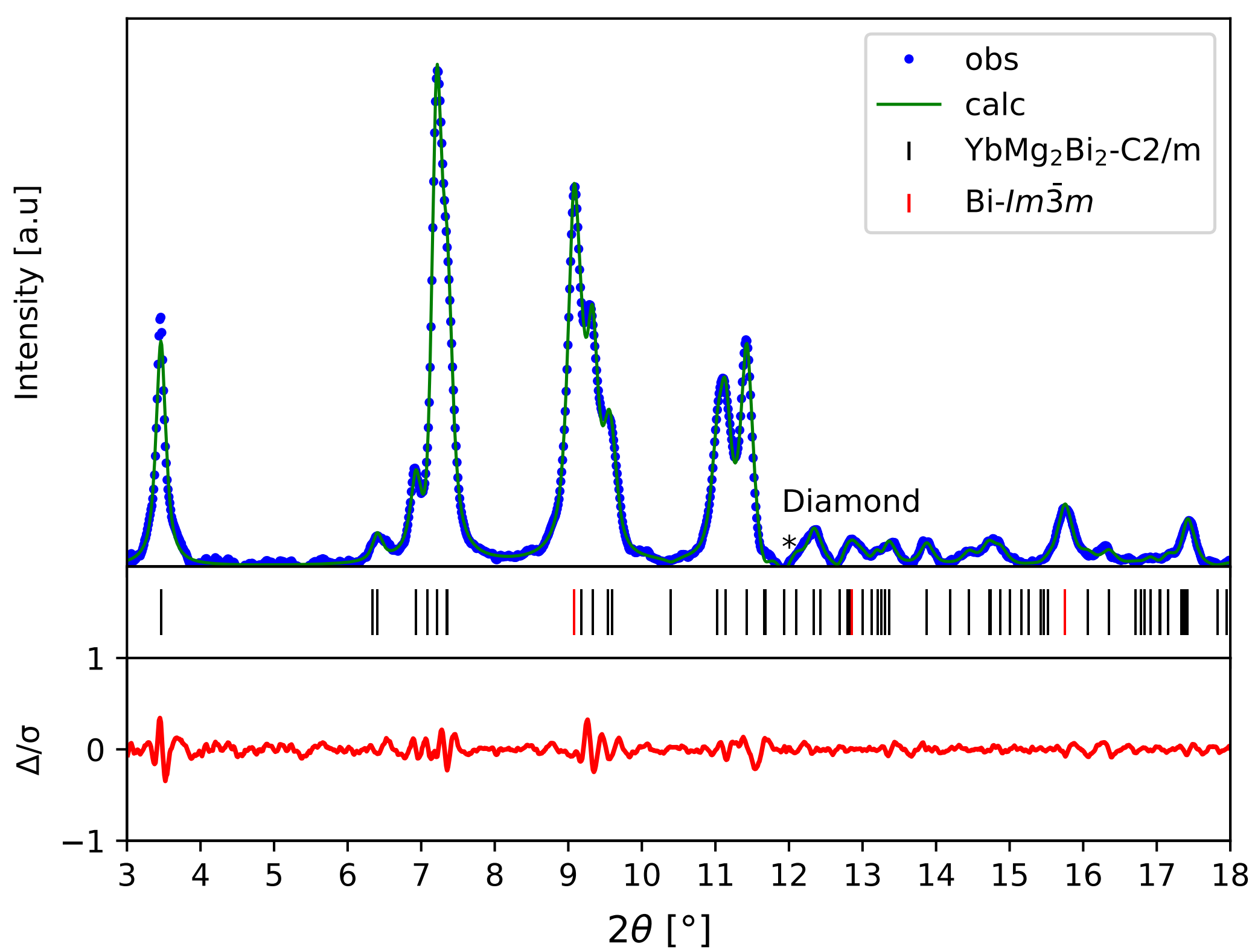

Figure S3: High-pressure ($C2/m$) Rietveld refinements for a) $CaMg_2Bi_2$ at 12.47 GPa, collected using λ = 0.434 Å (28.6 keV), and b) $YbMg_2Bi_2$ at 12.45 GPa, collected using λ = 0.413 Å (30.0 keV).

S4 Volume calculation of polyhedra and error estimations

The volumes of the octahedra and tetrahedra were calculated by treating them as regular polyhedra, using an average of the two distinct Bi-Bi bond distances as the edge length of the octahedron ($\mathrm{l_{oct}}$) and of the tetrahedron ($\mathrm{l_{tet}}$). The octahedron ($\mathrm{V_{oct}}$) and tetrahedron ($\mathrm{V_{tet}}$) volumes are then calculated as:

$$V_{oct} = \frac{\sqrt{2}l_{oct}^3}{3} \tag{S1}$$

and

$$V_{tet} = \frac{l_{tet}^3}{6\sqrt{2}} \tag{S2}$$

respectively. To calculate the uncertainty in the polyhedral volume, an error propagation scheme was used that starts with the uncertainty in the bond lengths as is explained next. For the octahedron volume, the longer Bi-Bi bond length ($\mathrm{l_{oct_I}}$) and the corresponding uncertainty of such bond ($\mathrm{u_{oct_I}}$) were used to determine the maximum octahedral volume $\mathrm{V_{oct_{max}}}$ as:

$$V_{oct_{max}} = \frac{\sqrt{2}\left(l_{oct_I} + u_{oct_I}\right)^3}{3} \tag{S3}$$

Similarly, the minimum octahedron volume $\mathrm{V_{oct_{min}}}$ is calculated using the shorter Bi-Bi bond length ($\mathrm{l_{oct_{II}}}$) and its associated uncertainty ($\mathrm{u_{oct_{II}}}$):

$$V_{oct_{min}} = \frac{\sqrt{2}\left(l_{oct_{II}} - u_{oct_{II}}\right)^3}{3} \tag{S4}$$

Likewise, the maximum tetrahedron volume ($\mathrm{V_{tet_{max}}}$) is calculated as a regular polyhedron via the longer Bi-Bi bond ($\mathrm{l_{tet_I}}$) and its uncertainty ($\mathrm{u_{tet_I}}$), as a modification of equation S2. It becomes:

$$V_{tet_{max}} = \frac{\left(l_{tet_I} + u_{tet_I}\right)^3}{6\sqrt{2}} \tag{S5}$$

Conversely, the minimum tetrahedron volume ($\mathrm{V_{tet_{min}}}$) is based on the smaller Bi-Bi bond that spans the polyhedron ($\mathrm{l_{tet_{II}} = l_{oct_I}}$) and its corresponding uncertainty ($\mathrm{u_{tet_{II}} = u_{oct_I}}$), which is then:

$$\mathrm{V_{tet_{min}}} = \frac{\left(\mathrm{l_{tet_{II}}} - \mathrm{u_{tet_{II}}}\right)^3}{6\sqrt{2}} = \frac{\left(\mathrm{l_{oct_I}} - \mathrm{u_{oct_I}}\right)^3}{6\sqrt{2}} \tag{S6}$$

which, again, is a modification of equation S2.

Now, in order to calculate the total polyhedral volume error ($\mathrm{e_V}$), the *symmetric* mean absolute percentage error is obtained with the following general relation:

$$e_V = \frac{1}{n}\sum_{i=1}^{n}\left|\frac{V_{avg} - V_i}{2\left(V_{avg} + V_i\right)}\right| \tag{S7}$$

where V_{avg} is the average volume and V_i is an extreme volume, i.e., either V_{max} or V_{min}. Then, using equation S7 the octahedral ($e_{V_{oct}}$) and tetrahedral volume ($e_{V_{tet}}$) errors become:

$$e_{V_{oct}} = \frac{1}{2}\left[\left|\frac{V_{oct} - V_{oct_{max}}}{2\left(V_{oct} + V_{oct_{max}}\right)}\right| + \left|\frac{V_{oct} - V_{oct_{min}}}{2\left(V_{oct} + V_{oct_{min}}\right)}\right|\right] \tag{S8}$$

and

$$e_{V_{tet}} = \frac{1}{2}\left[\left|\frac{V_{tet} - V_{tet_{max}}}{2\left(V_{tet} + V_{tet_{max}}\right)}\right| + \left|\frac{V_{tet} - V_{tet_{min}}}{2\left(V_{tet} + V_{tet_{min}}\right)}\right|\right] \tag{S9}$$

respectively.

S5 Equation of State Parameter Covariance and *F*–*f* Analysis

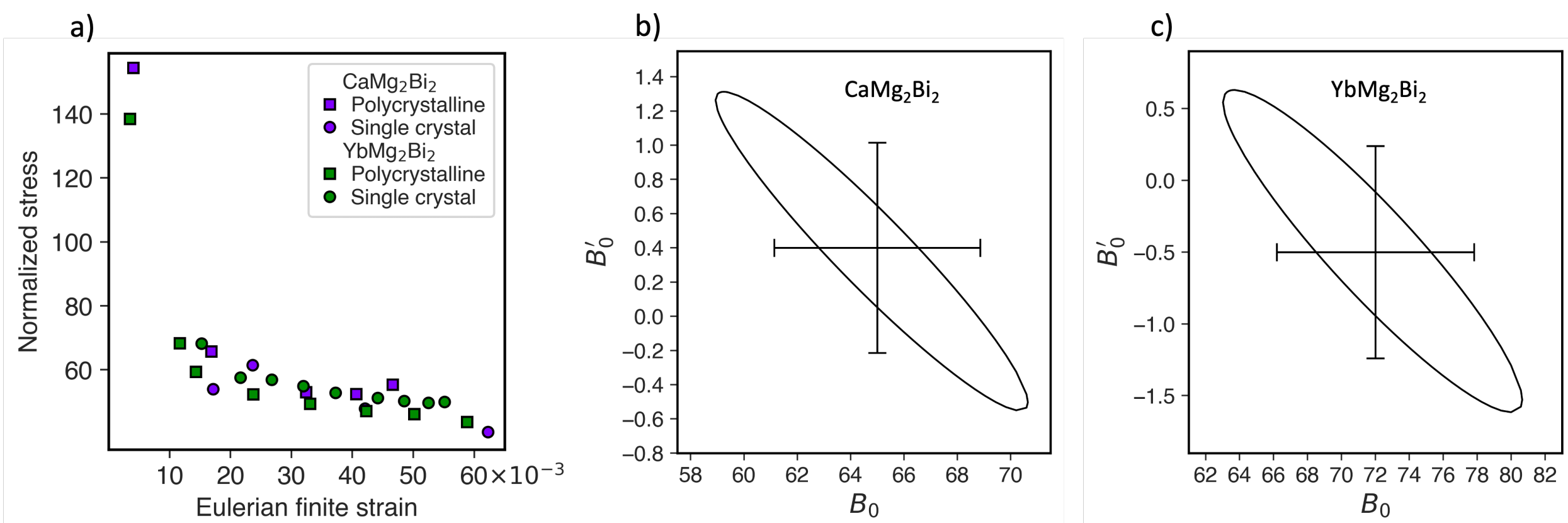

Figure S4: (a) Eulerian normalized pressure, F, as a function of Eulerian strain, f, for the trigonal phases of $CaMg_2Bi_2$ (magenta) and $YbMg_2Bi_2$ (green), calculated using the ambient-pressure volumes determined by single-crystal X-ray diffraction [1]. The negative best-fit slopes suggest $B_0' < 4$; however, third-order Birch–Murnaghan refinements yield strongly correlated values of B_0 and B_0', as illustrated by the 1σ and 2σ confidence ellipses, and produce B_0 values near 70 GPa together with low or negative values of B_0'. Because the independently measured ambient acoustic bulk moduli of 36 and 38 GPa [2] agree closely with the second-order results of 37(3) and 37.6(4) GPa, respectively, the second-order Birch–Murnaghan equation of state with $B_0' = 4$ was retained as the more stable constrained description of the compression data. Correlation between the bulk modulus, B_0, and its pressure derivative, B_0', obtained from third-order Birch–Murnaghan refinements for (b) $CaMg_2Bi_2$ and (c) $YbMg_2Bi_2$. The confidence ellipses illustrate the strong covariance between B_0 and B_0', while the error bars indicate the corresponding uncertainties in the fitted parameters.

S6 Pressure dependence of the lattice parameters and volume

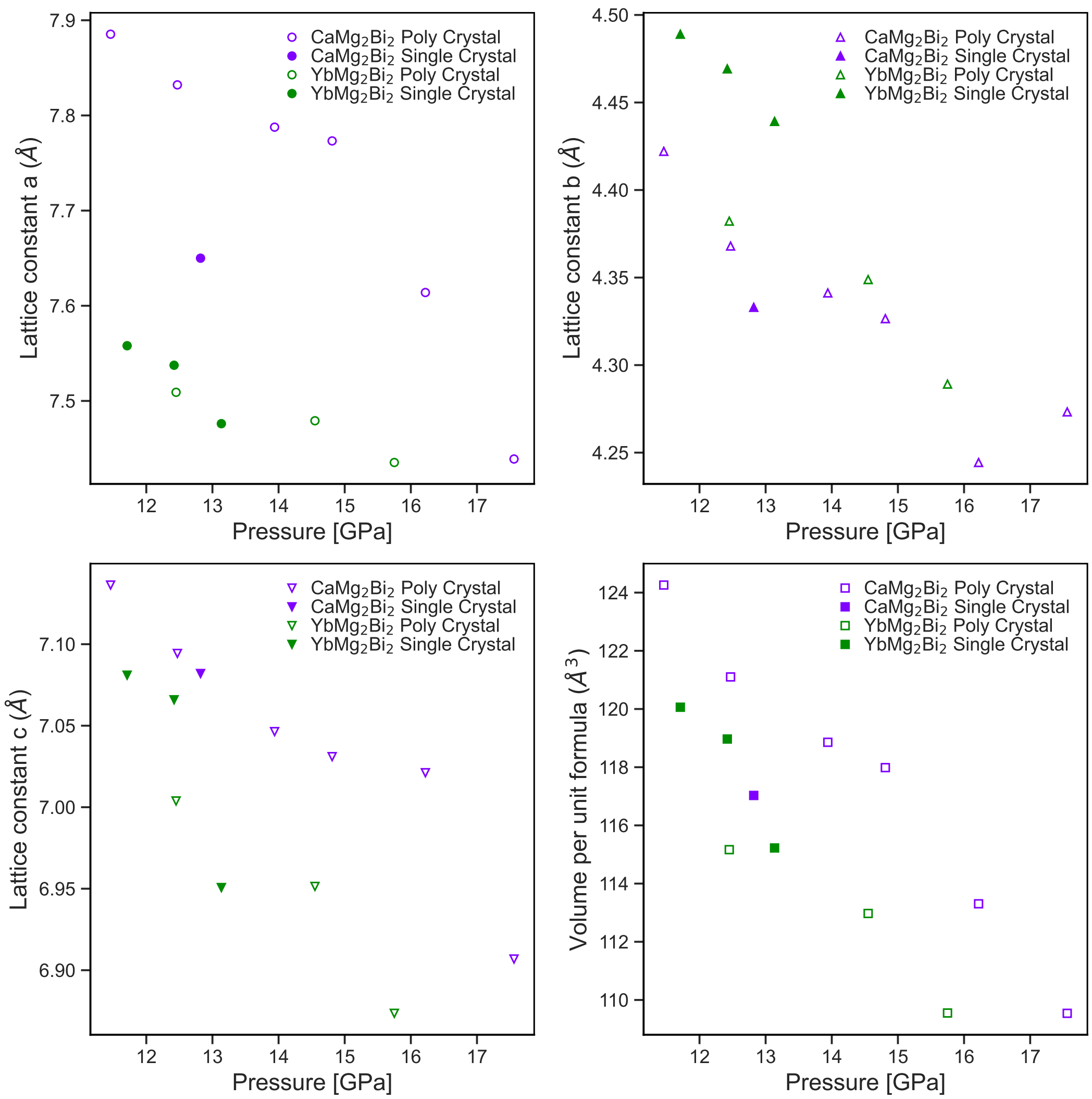

Figure S5: Pressure dependence of the lattice parameters and volume per formula unit of the high-pressure $C2/m$ phase of $CaMg_2Bi_2$ and $YbMg_2Bi_2$, based on the available single-crystal and polycrystalline data.

References

[1] Ross J Angel, Matteo Alvaro, and Javier Gonzalez-Platas. EosFit7c and a Fortran module (library) for equation of state calculations. *Z. Kristallogr. Cryst. Mater.*, 229(5):405–419, 2014.

[2] Wanyue Peng, Guido Petretto, Gian-Marco Rignanese, Geoffroy Hautier, and Alexandra Zevalkink. An unlikely route to low lattice thermal conductivity: Small atoms in a simple layered structure. *Joule*, 2(9):1879–1893, 2018.
